# How to improve the system of care for adolescents with emotional and behavioural problems from the perspective of care providers: a concept mapping approach

**DOI:** 10.1186/s12961-023-01096-1

**Published:** 2024-01-15

**Authors:** Lucia Bosakova, Zuzana Dankulincova Veselska, Daniela Filakovska Bobakova

**Affiliations:** 1 Department of Health Psychology and Research Methodology, Faculty of Medicine, P.J. Safarik University in Kosice, Tr. SNP 1, Kosice, 040 01 Slovakia; 2 Olomouc University Society and Health Institute (OUSHI), Palacky University in Olomouc, Univerzitni 22, Olomouc, 771 11 Czech Republic

**Keywords:** Children, Adolescents, Emotional and behavioural problems, System of care, Care providers, Concept mapping

## Abstract

**Background:**

Emotional and behavioural problems (EBP) are the most common mental health issues during adolescence, and their incidence has increased in recent years. The system of care for adolescents with EBP is known to have several problems, making the provision of care less than optimal, and attention needs to be given to potential improvements. We, therefore, aimed to examine what needs to be done to improve the system of care for adolescents with EBP and to assess the urgency and feasibility of the proposed measures from the perspective of care providers.

**Methods:**

We used Concept mapping, a participatory mixed-method research, based on qualitative data collection and quantitative data analysis. A total of 33 stakeholders from 17 institutions participated in our study, including psychologists, pedagogues for children with special needs, teachers, educational counsellors, social workers and child psychiatrists.

**Results:**

Respondents identified 43 ideas for improving of the system of care for adolescents with EBP grouped into 5 clusters related to increasing the competencies of care providers, changes at schools and school systems, support for existing services, transparency of the care system in institutions and public administration, and the adjustment of legislative conditions. The most urgent and feasible proposals were related to the support of awareness-raising activities on the topic of EBP, the creation of effective screening tools for the identification of EBP in adolescents, strengthening the role of parents in the process of care, comprehensive work with the family, creation of multidisciplinary support teams and intersectoral cooperation.

**Conclusions:**

Measures which are more accessible and responsive to the pitfalls of the care system, together with those strengthening the role of families and schools, have greater potential for improvements which are in favour of adolescents with EBP. Care providers should be invited more often and much more involved in the discussion and the co-creation of measures to improve the system of care for adolescents with EBP.

## Background

Adolescent mental health represents an important public health challenge [1, 2]. The estimated prevalence of mental health problems among adolescents is about 20% [3, 4], and up to 50% of all mental health conditions start before the age of 14 years [3]. Adolescent mental health problems and disorders thereby often persist throughout adolescence into adult life [5], have both short- and long-term impacts on health and represent a major burden of disease [6]. In addition, the coronavirus disease 2019 (COVID-19) pandemic has severely impacted the well-being of adolescents and has put them at an increased risk of various mental health problems [3]. Therefore, to reduce the burden of mental health problems, coordinated delivery of effective prevention and treatment is inevitable [7].

The most common mental health problems during childhood and adolescence are emotional and behavioural problems (EBP). They cover a range of problems that manifest themselves in different ways. Young people with such problems tend to be very lively in their speech, their moods change, they are often sad, they get angry quickly, they often engage in battles, they come into conflict with adults, they often skip school, bully classmates, drink alcohol, use drugs or steal [8–10]. Whether it is an emotional or behavioural issue, the same is true for both. These are problems that prevent adolescents from making full use of their potential, which could and should be fully developed during this period of their lives [11, 12].

The system of care for adolescents with EBP includes the full range of services provided, from parental support to counselling, psychological, social and psychiatric care. Although the systems of care might differ across countries, the need for improvements has been identified or recommended by various researchers and several countries are working to improve the performance of the care system and its ability to respond to client needs [11]. Previous research has shown that prevention, diagnosis and treatment in the system of care are currently suboptimal for a number of reasons. Uneven access and distribution of care [13, 14], inadequate identification of problems [15] and the postponement of care due to waiting lists [16] are the most common problems. Also, appointments with several experts extend the time from problem identification to the beginning of care [15], and stigmatization leading to denying the existence of problems and refusing of care by parents [17] play an important role. Further, insufficient organization of the system, leading to duplicate care [18], insufficient methodological guidance, high administrative burden and lack of institutional and personnel capacities [18–20] have been previously identified as barriers to optimal care for adolescents with EBP. As seen above, previous research has been predominantly focused on the identification of existing barriers within the system of care rather than solutions for its enhancement. Our study offers the possibility of filling the gap in existing knowledge in this area.

Improving the system of care in favour of adolescents with EBP requires going beyond its examination and a mere understanding of its pitfalls and barriers. It is also important to move to the next step and to focus on how to proceed further, with the aim to identify and prioritize specific proposals and measures that need to be implemented to improve the system of care for adolescents with EBP. The involvement of actors concerned with the issue of interest in the process of desired change might substantially increase the chances for success and a positive impact [21, 22]. Care providers in the system of care who are in daily contact with their clients and are familiar with their struggles and needs, have important insight into the care processes and understand how the system is organized, are among the most relevant sources of information, and their perceptions and proposals for improving the system of care might thus be of the great value. In addition, with the involvement of those concerned, the knowledge is created by those who could be, at the same time, a vital part of the implementation of such knowledge into practice, increasing the chances of its successful uptake [23]. In this paper, we focused and reported on the perspectives of care providers at different levels of work hierarchy from various institutions represented in the system of care. The aim of this study was to explore what needs to be done to improve the system of care for adolescents with EBP and to assess the urgency and feasibility of the proposed measures from the perspective of the care providers.

## Methods

This concept mapping (CM) study, focused on improvements in the system of care for adolescents with EBP, was not an individual study but was carried out as part of the bigger Care4Youth project – psychosocial development of adolescents with emotional and behavioural disorders in the system of care – a longitudinal study. This project aims to participatively map and improve the system of care for adolescents with EBP in close collaboration with care providers, parents/counsellors and adolescents themselves. Within the Care4Youth project, the triangulation of different research methods and data was used to increase the validity of the findings [24]. The CM study presented in this manuscript was preceded by a series of studies, such as a literature review (about the system of care for children with EBP), consultations with experts (both from academia and practice), network analysis (detailed mapping of institutions involved in the system of care and the links between them), a quantitative study (a prospective cohort study with adolescents with EBP, their parents/guardians, and care providers about health and health behaviour, family, school, peers, process and character of provided care) and a qualitative study (semi-structured interviews with care providers about the system of care, its setting and barriers).

### Theoretical framework

As our aim was to let care providers come up with their own ideas about potential measures that may improve the system of care for adolescents with EBP and not limit them to our preconceptions, we deliberately decided to avoid specifying any theoretical propositions or models at the outset of our investigation, in line with a more exploratory and data-driven approach. Once the data were collected and measures for improvement expressed by care providers, we compared their ideas with existing theoretical concepts to see the potential overlap.

### Design and setting

We used CM, an integrated mixed-method design based on qualitative data collection and quantitative data analysis, enabling a diverse group of stakeholders to qualitatively articulate their ideas and represent them in a variety of quantitatively derived results. CM is a method for assessing how study participants cluster their conceptual assessment of a particular topic by developing a conceptual framework with a visual display of the clustering [25]. It allows the mapping of complex concepts that are not explicitly identified by participants [26]. This method allowed us to apply a participatory approach, with stakeholders’ involvement and the empowerment of specific groups, such as front liners, and to visualize the results in a way accessible and understandable to various groups.

We conducted the CM study in the eastern part of Slovakia, with most of the participants providing care in Kosice, the second biggest city in Slovakia. In the selected area, a full range of institutions providing care to adolescents with EBP is available, and personal contacts of the research team in this area facilitated entry into the field. This helped us to include all types of care providers (from preventive counselling, social and healthcare) and to ensure a wide range of views.

### Sample

We recruited participants following Kane and Rosas [25] and Kane and Trochim [27], to ensure the availability of a wide variety of viewpoints and to support a broader range of people to adopt the resulting conceptual framework. We did this by involving a variety of actors in some way engaged in and/or responsible for the studied topic.

Based on our previous research in this area (literature review, network analysis), consultations with experts from the field and referrals, we identified three categories of care providers within the system of care for adolescents with EBP in Slovakia: preventive counselling, social care and mental healthcare. Preventive counselling care primarily solves problems in adolescents that are associated with the school environment and includes mainly schools (teachers, school psychologists, educational counsellors, pedagogues for children with special needs), centres of pedagogical–psychological counselling and prevention, and centres of special pedagogical counselling (clinical psychologists, pedagogues for children with special needs, educational counsellors). Social care is predominantly represented by the Office of Labour, Social Affairs and Family and non-profit organizations collaborating with state institutions (clinical psychologists, social workers), which are supposed to prevent crises in families, protect the rights and interests of children, and prevent a deepening and repeating of disorders of healthy development, including mental, physical and social areas of development. Mental healthcare includes outpatient clinics of clinical psychologists for children, child psychiatrists, psychiatric hospitals and sanatoriums (clinical psychologists, child psychiatrists, social workers) and provides a broad spectrum of services, such as social counselling, psychological counselling, therapy and psychiatric care for children and adults.

Subsequently, the purposive sampling technique was used to recruit stakeholders of different work-level hierarchies across the identified three main categories, ensuring that all types of professionals in our region working in the system of care for adolescents with EBP will be represented in our sample. We initially addressed 40 stakeholders, of which 33 agreed to participate in the study (82.5% response rate). Those who refused to participate were mainly from top managerial positions and declined to participate due to their excessive workload. Participants of our study were from 17 various institutions and included psychologists, child psychiatrists, social workers, pedagogues for children with special needs, teachers, and educational counsellors. All participants were female (100%) given the highly feminized sectors in which the study was conducted, with ages ranging from 25 to 65 years.

As it is typical with concept mapping phase approaches, not all participants took part in all phases [28]. Twenty-five participants were able to attend an in-person 1 day workshop, where brainstorming together with sorting/rating took place. The remaining eight participants, who agreed to participate in the study but were unable to attend the workshop, were addressed again, they checked and approved the brainstormed items and performed the sorting/rating individually. Also, twenty-three participants ultimately took part in the interpretation session, but with a balanced representation of all three categories of care. The sample size for each CM step in our study was sufficient to meet the requirements for valid and reliable results [29]. All participants were provided with comprehensive information about the study and gave their written consent. Table 1 provides an overview of the number and type of participants (and institutions) based on their participation in particular phases of the CM study (brainstorming, sorting/rating, interpretation).

**Table 1:** Number and type of participants of the CM study

	Preventive counselling care			Social care			Healthcare		
CM step	B	S/R	I	B	S/R	I	B	S/R	I
Number of institutions	7	7	7	6	7	6	4	5	5
Number and type of providers	10	11	9	10	16	8	5	6	6
Psychologist	5	6	4	4	5	3	2	2	2
Child psychiatrist	–	–	–	–	–	–	2	3	3
Social worker	–	–	–	6	11	5	1	1	1
Pedagogue for children with special needs	2	2	2	–	–	–	–	–	–
Teacher	2	2	2	–	–	–	–	–	–
Educational consultant	1	1	1	–	–	–	–	–	–

*B* brainstorming, *S/R* sorting and rating, *I* interpretation

### Procedure and analysis

CM activities were carried out from November 2018 to November 2019. The procedure consisted of five steps: (1) preparation, (2) brainstorming, (3) sorting and rating, (4) analysis and (5) interpretation, as suggested by Kane and Rosas [28].

In the preparation step (1), we identified a focus prompt (the core question to be asked) and held a pilot of the CM session with the broader research team (also researchers not included in this study) to discuss the appropriateness of the focus prompt formulation, to formulate possible statements and to discuss the facilitation process to offer suggestions for improving the subsequent brainstorming session.

Steps (2) and (3) (brainstorming, sorting and rating) were conducted together in person during the 1 day workshop. In the brainstorming session (2), we first presented the aim of the study and a brief introduction to the CM method. We then presented the focus prompt:

“What do you think needs to be done to improve the system of care for adolescents with emotional and behavioural problems in their favour?”

*[Čo treba podľa Vás spraviť, aby sa zlepšil systém starostlivosti o dospievajúcich s emocionálnymi problémami a problémami v správaní v ich prospech?]*

We further explained and defined what is meant by the “improvement of the system of care” and what is meant by the “in their (children’s) favour”, to ensure that participants had a solid understanding of the issue. Subsequently, we divided the participants into four smaller subgroups of four to seven participants: the preventive counselling care subgroup, the social care subgroup, the healthcare subgroup and the subgroup of managers. The first three subgroups consisted of front-line employees, whereas the fourth subgroup consisted of managers from all the above-mentioned fields. The latter subgroup was created to minimize the impact of power relations. Subsequently, participants in all subgroups were asked to respond to the focus prompt and generate as many statements as they wish. Each subgroup had its own facilitator, who visibly wrote generated statements on the flipchart, and a research assistant who recorded the generated items into an online shared folder. An additional research assistant managed the online shared folder and inputs from subgroup research assistants. This session lasted approximately 1.5 hour until data saturation was achieved and no new ideas were generated within the brainstorming. Next, the research team, together with all the participants, removed obvious redundancies and overlapping concepts and merged those that were semantically similar into a reduced, parsimonious set of statements. This session was facilitated by the main facilitator and lasted approximately 2 hours. From the original 80 statements, a representative list of 43 statements (master list) was created to conduct the following procedure.

In the sorting and rating session (3), we printed all 43 statements individually on small cards and gave a complete set of cards to the participants. We asked them to individually sort the cards (statements) into piles that make sense to them and to create a label for each pile. We explained three restrictions of this activity: (a) a card may only be placed in one pile at a time, (b) each card may not be alone in its pile and (c) all cards may not be grouped in the same pile. Subsequently, we asked them to rate these statements according to two selected domains of interest – urgency and feasibility (Likert scale: 1, not urgent or low feasibility; 4, very urgent or high feasibility). These sessions lasted together approximately 2 hours.

In the analytical step (4), before the statistical analyses, a quality review of the data obtained in sorting and rating was performed to exclude those participants who did not follow the sorting and/or rating guidelines, did not complete at least 75% of the task or who provided negligent answers [27]. Data from all 33 participants in the sorting and rating step passed the quality review and were analysed using groupwisdom software. Sorting data were analysed using multidimensional scaling to generate a point map, where the statements were plotted based on the number of times participants grouped them together, with those that were frequently grouped together positioned close to each other. Hierarchical cluster analysis was conducted to generate cluster maps, where the statements were aggregated into clusters based on their proximity to each other on the point map [30]. The findings of this analysis were discussed with the research team, following the CM methodology [25]. The research team chose a varying maximum number of clusters (2–10, that is, the highest and the lowest desired number of clusters, as sorted by participants) and discussed the final cluster solution. The research team clustered the measures in various ways, checking for the stress index (the metric indicating the degree to which a multidimensional scaling solution fits the original similarity matrix), but also reviewing contents both qualitatively, and also by using the bridging/anchoring analysis – and all this while keeping in mind the focus prompt and the project objectives. The bridging/anchoring analysis shows the relationship of a statement to its location on the map, based on how it was sorted with other statements, with “anchors” being those statements that were sorted often with the items that surround them which conceptually anchor that area of the map [27]. To confirm this analysis, the group also performed a spanning analysis to visualize the statements’ strength of connection to every other item on the map (the more the selected item was sorted with each other item on the map, the thicker the connection line between them) [27]. After a review of the content and alignment, the research team proposed five cluster solutions, that could support the desired outcomes of the project and would be understandable and interpretable for the participants, but which were also most frequently used by the participants themselves during the sorting phase (modus). To inform potential priority areas of action, we further identified statements from a “Go zone”, that is, rated as the most urgent and feasible. Other quadrants within the importance and feasibility plots visualize statements that may be less likely to mobilize action because of lower ratings, as well as statements rated as highly important but low on feasibility, which may expose barriers that may prevent action on critical factors [28].

Finally, the outcomes of the analyses (a five-cluster solution cluster point map, rating maps and Go zone map) were discussed within the interpretation workshop (5). The interpretation group consisted of 23 stakeholders who participated in the previous steps of the CM (9 from preventive care, 8 from social care and 6 from health care, see Table 1). During this in-person workshop, the interpretation group of participants was asked to agree on the final five-cluster solution, review the groups of statements, and discuss and finalize the cluster labels. Finally, participants discussed also the set of priority statements from the Go zone.

## Results

### Clusters of measures related to improving the system of care for adolescents with EBP

We obtained a five-cluster solution approved by the interpretation group as follows:

Cluster 1. Increasing the competencies, possibilities and opportunities for providers and institutions in the system of care.

Cluster 2. Changes at the level of the school and the school system.

Cluster 3. Support for existing services targeting children and families.

Cluster 4. Increasing the transparency and functionality of the system of care at the level of institutions and public administration.

Cluster 5. Modification and creation of legislative conditions in the system of care for children with EBP.

**Fig. 1:**
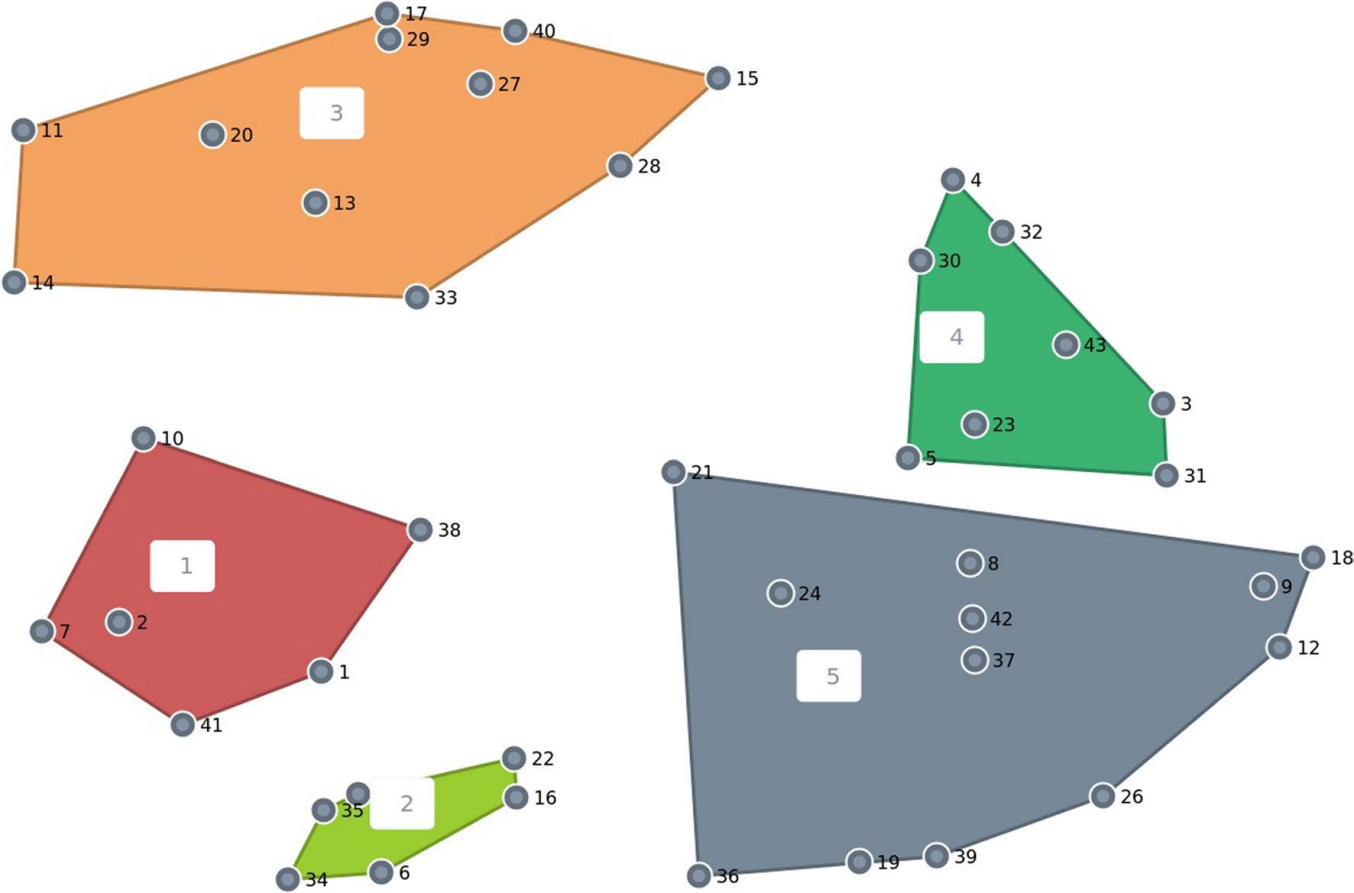
Clusters of measures: point map – final five-cluster solution. Each dot represents a single measure proposed by participants. The closer the dots are to each other, the more participants sorted the measures into the same piles, thus are more likely to regard similar concepts. The size of the surface of a cluster indicates the degree to which its various contributing items are related

The cluster point map is shown in Fig. 1. In this map, a point (dot) represents one specific measure suggested by participants, and the distance between the points indicates the likelihood that participants have placed the measures concerned in the same group; the clusters represent discrete groupings of related measures. The stress index was 0.2334, suggesting a strong fit between the cluster map and the data (typically, the stress index in CM studies should be between 0.10 and 0.35 in accordance with Kane and Rosas [25]).

### Rating of clusters by urgency and feasibility

**Fig. 2:**
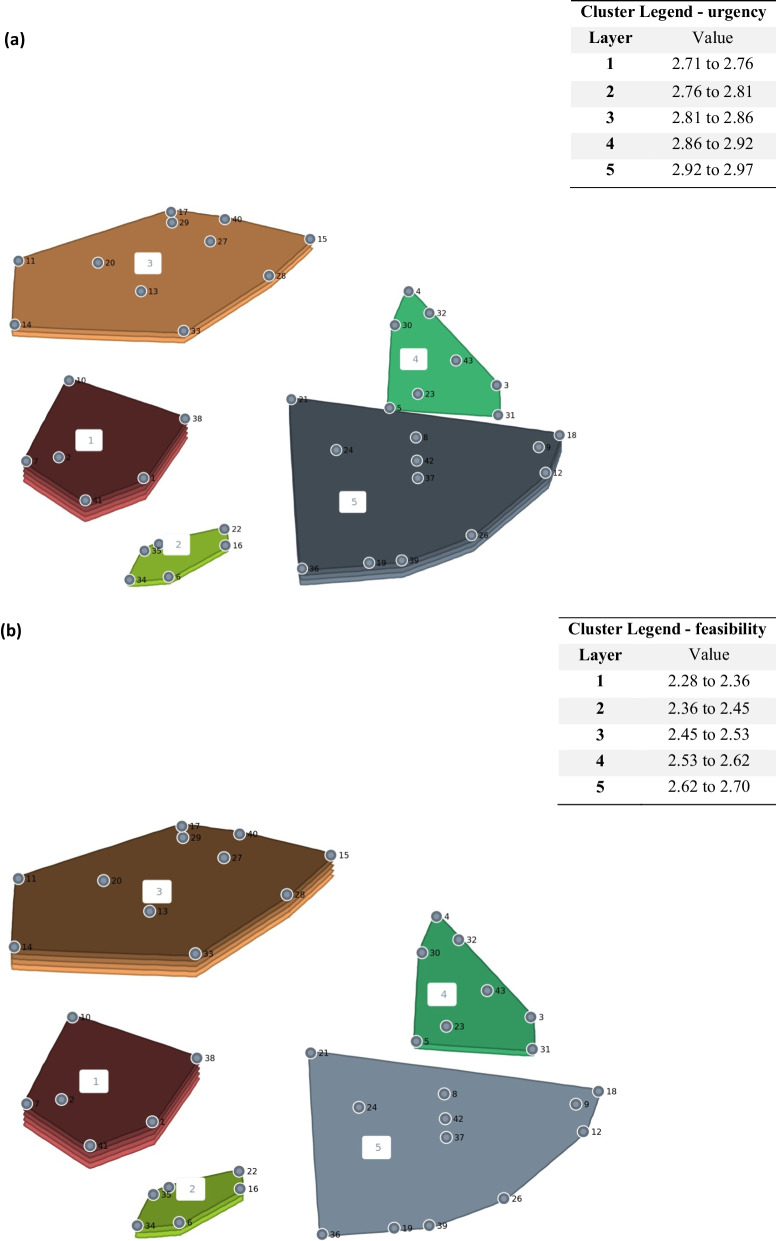
Urgency (**a**) and feasibility (**b**) of measures per cluster: cluster rating maps. More layers indicate more urgency and feasibility.

The urgency and feasibility of the various clusters as rated by participants are shown in the cluster rating maps (Fig. 2), where the third dimension (layer) displayed on top of the clusters represents the mean ratings of the selected criteria (urgency; feasibility) across all items, while the number of layers represents the higher or lower mean ratings related to other clusters on the map.

As regards urgency, participants considered cluster 1, related to increasing the competencies, possibilities and opportunities for providers and institutions in the system of care, as the most urgent. Cluster 4, related to the transparency and functioning of the system of care at the level of institutions and public administration, was rated by the participants as the least urgent.

In terms of feasibility, cluster 1, related to increasing the competencies, possibilities and opportunities for providers and institutions in the system of care, and cluster 3, related to support for existing child and family services, were rated by participants as the most feasible. On the other hand, cluster 5, related to the modification and creation of legislative conditions in the system of care for children with EBP, was rated by the participants as the least feasible.

Regarding both the urgency and feasibility, a match occurred in cluster 1, which was seen to be both very urgent and highly feasible. We found the biggest difference in cluster 5, which was seen by participants as very urgent, but the least feasible. Table 2 shows the ranges of urgency and feasibility per cluster.

**Table 2:** Urgency and feasibility of measures per cluster: mean scores and ranges

	Cluster	1	2	3	4	5
Number of measures		6	6	11	8	12
Urgency	Mean (SD)	2.97 (0.13)	2.79 (0.28)	2.83 (0.26)	2.71 (0.26)	2.86 (0.39)
	Range (min–max)	2.84–3.15	2.45–3.27	2.55–3.42	2.24–3.09	2.24–3.64
Feasibility	Mean (SD)	2.67 (0.31)	2.45 (0.14)	2.70 (0.40)	2.42 (0.42)	2.28 (0.40)
	Range (min–max)	2.21–3.09	2.30–2.67	1.84–3.21	1.88–3.15	1.45–2.85

Mean values per cluster—higher scores indicate more urgency and more feasibility; Minimum and maximum values of measures per cluster, the possible range of values was 1–4

*SD* standard deviation

### Rating of individual measures by urgency and feasibility

**Fig. 3:**
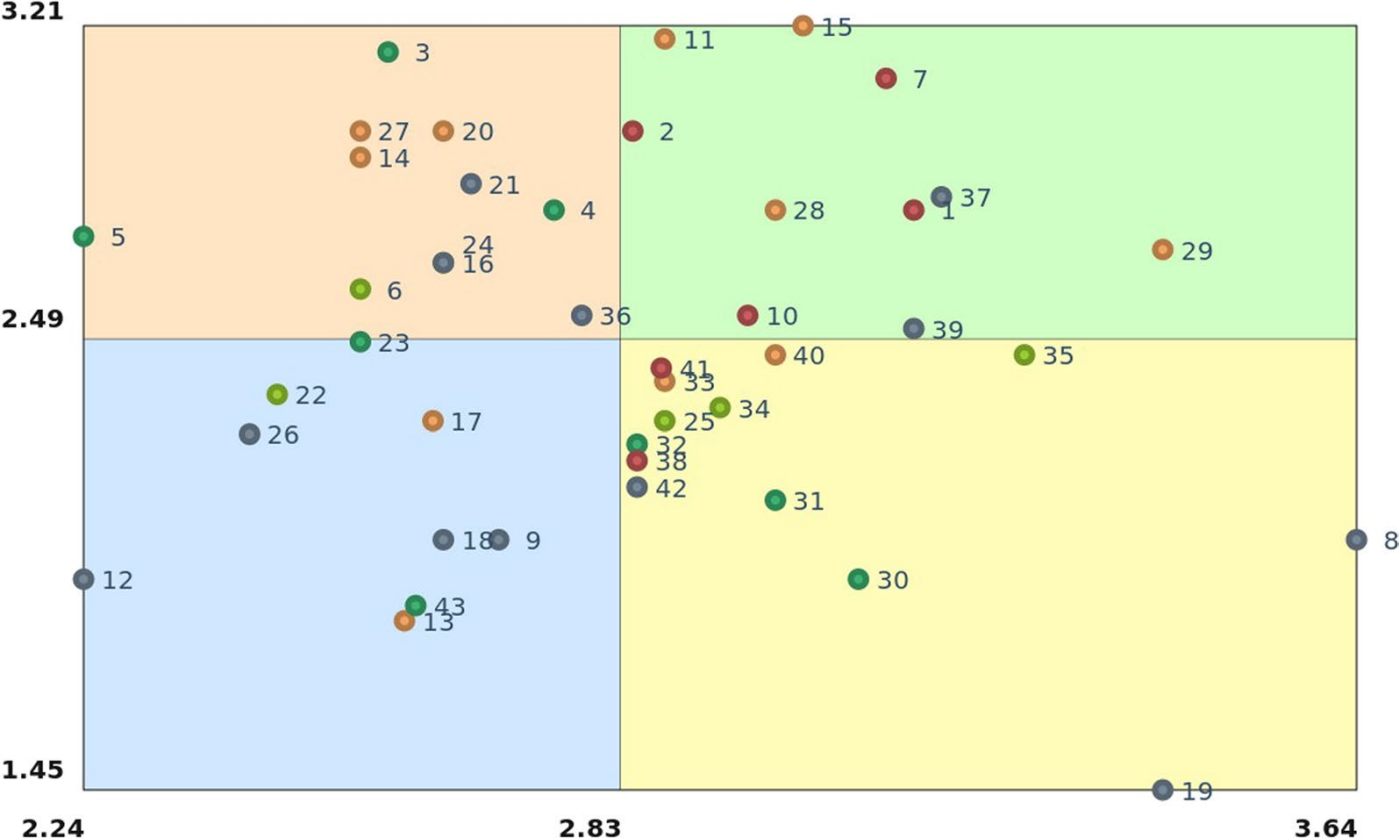
Rating of individual measures by urgency and feasibility: Go zone map. Each dot represents a measure. The *x* axis shows the range of mean values for urgency (2.24–3.64); the *y* axis shows the range of mean values for feasibility (1.45–3.21). Dots in the green upper-right quadrant indicate measures that were rated above the mean (most urgent and feasible).

In the Go zone map (Fig. 3) the priority measures rated as the most urgent and the most feasible are placed in the green sector in the upper-right corner. Out of 43 proposed measures, 10 were rated as the most urgent and most feasible and should be, according to the participants, implemented with a priority to improve the system of care for adolescents with EBP.

Priority measures belong to only three out of five clusters. The highest number of priority measures belong to cluster 1 (increasing the competencies, possibilities and opportunities for providers and institutions in the system of care) and cluster 3 (support for existing child and family services). All individual priority measures divided by clusters are listed in Table 3.

**Table 3:** Individual measures rated as the most urgent and feasible (upper-right quadrant from Go zone) divided by clusters: Go zone

Cluster number	Measure number	Measure	Urgency range [2.84–3.18] Median 3.08 SD 0.16	Feasibility range [2.52–3.21] Median 2.81 SD 0.23
			Mean	Mean
1	7	Development of skills and supervision of experts, pedagogical and professional workers for work with children and adolescents with EBP	3.12	3.09
	1	Creation of a professional team – school psychologist, social pedagogue, special pedagogue, school social worker, who would be regular employees of the counselling facility, to have the necessary competencies (in accordance to the needs of the particular school)	3.15	2.79
	10	Creation of an effective screening tool for EBP in children and adolescents (tool for early detection of the problem for teachers, psychologists, educational counsellors, school special pedagogues and so on)	2.97	2.55
	2	Introduction of the services for care providers, such as individual supervision, Balint groups, psychotherapy as prevention of burnout	2.84	2.97
3	29	Work comprehensively with the family (for example, in children and family centres)	3.42	2.70
	28	Support of outreach service (bring service to clients in case that family does not have the capacity to look for it)	3.00	2.79
	15	Media campaign – detabooing the topic	3.03	3.21
	11	Educating parents about EBP in children and adolescents and about developmental milestones and factors that can affect healthy development (information leaflets, brochures)	2.88	3.18
5	37	Improvement of the implementation of existing measures (psychological and special–pedagogical)	3.18	2.82
	39	Adjustment of legislation for school competencies in solving the crisis and special situations	3.15	2.52

## Discussion

This study aimed to explore what needs to be done to improve the system of care for adolescents with EBP, and to assess the urgency and feasibility of the proposed measures from the perspective of the care providers. Participants proposed 43 measures sorted into 5 distinct clusters, with cluster 1, related to increasing the competencies, possibilities and opportunities for providers and institutions in the system of care, being the most urgent and feasible. The biggest difference in terms of urgency and feasibility was found in cluster 5, related to the modification and creation of legislative conditions in the system of care for children with EBP, which was seen by participants as very urgent, but the least feasible. Overall, ten individual measures in the Go zone were rated as the most urgent and feasible and should be implemented with priority to improve the system of care for adolescents with EBP. The proposed priority measures covered a variety of topics, from inducing changes in societal discourse to improving access to care for clients, and strengthening the competencies of care providers, families and schools.

To improve the system of care for adolescents with EBP, participants suggested measures which are in line with general ecological system theories [31] but also with specific theoretical models of access to and organization of psychosocial care and barriers associated with it [32–35]. These models differentiate the main levels that should be taken into account – society (macrosystem), care system (exosystem) and care provider and client (mesosystem and microsystem). Cluster 1 covers topics related to increasing competencies at the personal and organizational level, together with the improvement of the working conditions of care providers which perfectly fits into Brofenbrenner’s exosystem, typical for links between social settings that do not involve the child directly but have a huge impact on it. Cluster 2 focuses on strengthening the role of schools which are typically in theoretical models part of the children’s immediate environment – the microsystem [31]. Cluster 3 also mirrors the immediate surroundings of the child—his/her microsystem [31], and is related to improvement of the availability and quality of care provided for the family. Clusters 4 and 5 are combinations of exosystems and macrosystems (refers to the already established society and culture in which the child is developing), with cluster 4 related to increasing the efficiency of provided care on the organizational and regional level, and cluster 5 rather on the governmental level. Overall, the proposed solutions are rather focused on streamlining the provided care, with most measures directed towards the wider societal context and system of care with care providers, and transfer responsibility for the improvement in care to the recipients of care only to a minimal extent.

We further found that cluster 1, related to increasing the competencies, possibilities and opportunities for providers and institutions in the system of care, was rated as the most urgent and feasible. These are the measures that are directly related to care providers, therefore this result might reflect their effort to actively participate in changes to improve the care system. Similar opinions were expressed by care providers also in previous qualitative research [11, 18, 36, 37], with further education, training, supervision and overall workforce development as crucial for the provision of optimal care. We found the biggest difference in cluster 5, related to the modification and creation of legislative conditions in the system of care for children with EBP, as it was rated as very urgent but least feasible. The explanation of our results about the low feasibility of measures connected with the modification and creation of legislative conditions could be also found in previous qualitative research based on the perspectives of care providers [11, 18, 36]. The first explanation might be that changes regarding the system of care usually span several ministries, with the cooperation that is needed for successful legislative change considered rather problematic [11, 18]. Second, if changes are not accompanied by precise allocation of financial resources and the personal capacities needed for such change, their implementation is limited [11, 36]. Third, changes often do not reflect the reality and needs articulated by people in practice, resulting in low feasibility due to the fact that suggested changes are not practice-based. And fourth, practice-based changes are perceived to need lobbying which may take years [11]. Thus, we may hypothesize that care providers might perceive little or no control over the legislative conditions, as these can only be changed through political processes. Overall, measures perceived as those in the hands of care providers were considered as the most feasible, while measures perceived to be in the hands of legal bodies were seen as the least feasible. Nevertheless, the high urgency of cluster 1 and cluster 5 suggests that the improvement of the system of care for adolescents with EBP is in great need of both removal of known barriers for efficient legislative change as well as the creating of space for individual active change by care providers themselves.

We also found ten priority measures which were rated by participants as the most urgent and feasible. Proposed measures cover topics emerging from societal discourse (measure 15) to main actors, namely care providers (measures 7, 1, 10, 2, 37), family (measures 29, 28, 11, 37) and school (measures 1, 10, 37, 39). This is in accordance with Levesque’s conceptual framework of access to healthcare [35] which “identifies relevant determinants that can have an impact on access from a multilevel perspective where factors related to health systems, institutions, organisations and providers are considered with factors at the individual, household, community, and population levels”. The Go zone indicates the complementarity and top-down direction of instant solutions that start with the need for a media campaign to detaboo the topic of EBP. Such a solution might help to raise awareness and lower the stigma towards a change in the societal discourse, which was found in previous research to be among the barriers to optimal care [7, 8, 17]. Fear of stigmatization and/or previous negative experience by adolescents and their parents influence their access to and use of psychosocial care, as well as their attitude towards the system of care [38, 39]. Further, increasing the competencies of care providers together with the improvement of their working conditions are crucial to improve the system of care for adolescents with EBP, as repeatedly stated in previous qualitative research by care providers themselves [11, 18, 36, 37]. Care providers also articulated the necessity of further collaboration with schools via strengthening their competencies and their role in the system of care for children with EBP, as these are the institutions that play an essential role in adolescents’ lives [11]. Schools may not be only the ideal place for the early detection of a problem, but should also be the place for early professional intervention [40]. Finally, part of the proposed measures focused on the families themselves and suggested the need for efficient outreach systems that would be able to bring care closer to them. Efficient outreach should be followed by comprehensive workflows focusing on the family as a whole, educating parents and increasing their awareness, resulting in informed and empowered parents who were recognized as major enablers of optimal care by previous research [18].

In general, most of the proposed measures focus on increasing the availability and quality of care provided and target its barriers without putting a burden on recipients of care by suggesting an increase in their abilities to engage with a system of care as it is. Given that children with EBP often come from families with multiple issues [41, 42], it can be seen as appropriate that most of the proposed measures aim to ensure that the care system can respond to the barriers of recipients rather than transfer the responsibility to them. At the same time, these results indicate that our participants perceive critically the quality of the current system of care in which they have participated, and came up with relevant proposals on how to change the system itself in favour of availability and quality for their clients.

### Strengths and limitations

We consider the strongest aspect of this study to be the opportunity to give care providers a voice and empowerment. Also, the CM methodology used is worth mentioning as it enables a sense of commitment to be given to everyone involved, which increases the chances of successful implementation of the study results. Another strength of this study is the quality of the data. This CM study was preceded by a series of steps (literature review, consultations with experts, network analysis, quantitative research with children, parents/guardians, care providers and qualitative research with care providers) which enabled us to (1) ensure thorough preparation of the CM study, that is, set the most accurate focus prompt and not omit key players; (2) gain rapport with stakeholders and thus limit socially desirable responses in the CM study and to increase their commitment; and also (3) increase the validity of CM study results, as findings from previous steps provide means for triangulation of evidence as obtained in CM. However, it is also necessary to mention some limitations. The CM methodology used might be prone to social desirability, although we have eliminated this risk, for example, by creating the subgroup of managers during the brainstorming to minimize the impact of power relations. Also, women more often participated in this study than men, which may possibly have an impact on the findings. Moreover, the participation of some types of stakeholders, such as public health authorities or care providers from the private sector, was relatively limited. Including more of these actors could have strengthened the findings of this study.

### Implications

Our study implies that, regarding research, the next step should be to thoroughly analyse the Gap zone, that is, the quadrant that visualizes statements rated as highly important but low on feasibility, which may expose barriers that could prevent action on critical aspects. It is also necessary to perform additional CM studies with parents and adolescents with EBP themselves, complemented by qualitative in-depth interviews with these actors, as this might also add to efforts to improve the system of care for adolescents with EBP.

We believe this study is a summary of ready-to-go policy suggestions that can be immediately put into practice by policy-makers at various levels of governance. To improve the system of care for adolescents with EBP, care providers propose several measures. From the point of view of care providers, measures aimed at removing barriers in the system (facilitating access to care provided, increasing the quality of care provided) are more effective than measures that place the burden of responsibility on the shoulders of care recipients. Although the involvement of care recipients and their families is extremely important, it should be, however, done in a sensible way, by seeking and strengthening their internal and external sources of support and resilience. The unifying element that has the potential to bring the provided care closer to recipients of care and their families is the school. This could be based on strengthening the professional and personnel capacities of all involved – teachers and educators for working with adolescents with EBD and their families, and professionals for cooperation with teachers and educators. In general, measures that are directly in the hands and competence of care providers are the most feasible, while measures that require government intervention and legislative changes are the least feasible. Therefore, government support, as well as the removal of bureaucratic barriers, would be very welcomed by care providers. In summary, measures that are more accessible and responsive to the pitfalls of the care system, together with those strengthening the role of families and schools, have greater potential for improvements that are in favour of adolescents with EBP. The suggestions and experiences of the providers are based on their daily practice and represent a valuable source of information. Therefore, care providers should be much more invited and involved in the discussion and co-creation of measures to improve the system of care for adolescents with EBP.

## Conclusions

To improve the system of care for adolescent with EBP, several measures were suggested by respondents. Based on our study, it could be concluded that measures that are more accessible and responsive to the pitfalls of the care system, together with those strengthening the role of families and schools have greater potential for improvements which are in favour of adolescents with EBP. Care providers should be much more invited and involved in the discussion and co-creation of measures to improve the system of care for adolescents with EBP.

## Data Availability

Datasets generated and/or analysed during the current study are available from the corresponding author upon reasonable request.

## Abbreviations

EBP: Emotional and behavioural problems
CM: Concept mapping

## References

[1] Patton GC, Sawyer SM, Santelli JS, Ross DA, Afifi R, Allen NB (2016). Our future: a lancet commission on adolescent health and wellbeing. Lancet.

[2] Ravens-Sieberer U, Ottová-Jordan V, Matthes M (2016). Children’s mental health in Europe: the current situation and its implications. Improving the quality of childhood in Europe.

[3] Guidelines on mental health promotive and preventive interventions for adolescents. Geneva: World Health Organization (WHO); 2020. https://apps.who.int/iris/bitstream/handle/10665/336864/9789240011854-eng.pdf. 33301276

[4] Alemán-Díaz AY, Backhaus S, Siebers LL, Chukwujama O, Fenski F, Henking CN (2018). Child and adolescent health in Europe: monitoring implementation of policies and provision of services. Lancet Child Adoles Health.

[5] Ormel J, Raven D, van Oort F, Hartman CA, Reijneveld SA, Veenstra R (2014). Mental health in Dutch adolescents: a TRAILS report on prevalence, severity, age of onset, continuity and co-morbidity of DSM disorders. Psychol Med.

[6] Mokdad AH, Forouzanfar MH, Daoud F, Mokdad AA, El Bcheraoui C, Moradi-Lakeh M (2016). Global burden of diseases, injuries, and risk factors for young people’s health during 1990–2013: a systematic analysis for the Global Burden of Disease Study 2013. Lancet.

[7] GBD 2019 Mental Disorders Collaborators (2022). Global, regional, and national burden of 12 mental disorders in 204 countries and territories, 1990–2019: a systematic analysis for the Global Burden of Disease Study 2019. Lancet Psychiatry.

[8] Achenbach TM, Rescorla, Leslie. Manual for the ASEBA school-age forms & profiles: an integrated system of multi-informant assessment. Open WorldCat. Burlington: ASEBA; 2001.

[9] Achenbach TM, McConaughy SH (1997). Empirically based assessment of child and adolescent psychopathology: practical applications.

[10] Wadsworth ME, Hudziak JJ, Heath AC, Achenbach TM (2001). Latent class analysis of child behavior checklist anxiety/depression in children and adolescents. J Am Acad Child Adolesc Psychiatry.

[11] Dankulincova Veselská Z, Bosáková L, Fiľakovská Bobáková D, Husárová D, Kopčáková J. Labyrinth journey: Adolescents with emotional and behavioral problems in the care system [Cesta labyrintom: Dospievajúci s emocionálnymi problémami a problémami v správaní v systéme starostlivosti.]. Košice: Vydavateľstvo ŠafárikPress; 2020.

[12] Ross DA, Hinton R, Melles-Brewer M, Fogstad H, Banerjee A, Mohan A (2020). Adolescent well-being: a definition and conceptual framework. J Adolesc Health.

[13] Paclikova K, Dankulincova Veselska Z, Madarasova Geckova A, van Dijk JP, Reijneveld SA (2020). Adolescent enrollment in psychosocial care: do parents make a difference?. Int J Environ Res Public Health.

[14] Garland AF, Lebensohn-Chialvo F, Hall KG, Cameron ERN (2017). Capitalizing on scientific advances to improve access to and quality of children’s mental health care. Behav Sci Law.

[15] Reijneveld SA, Brugman E, Verhulst FC, Verloove-Vanhorick SP (2004). Identification and management of psychosocial problems among toddlers in dutch preventive child health care. Arch Pediatr Adolesc Med.

[16] Oruche UM, Downs S, Holloway E, Draucker C, Aalsma M (2013). Barriers and facilitators to treatment participation by adolescents in a community mental health clinic. J Psychiatr Ment Health Nurs.

[17] Bowers H, Manion I, Papadopoulos D, Gauvreau E (2012). Stigma in school-based mental health: perceptions of young people and service providers. Child Adolesc Mental Health.

[18] Paton K, Hiscock H (2019). Strengthening care for children with complex mental health conditions: views of Australian clinicians. PLoS ONE.

[19] Pfefferle SG (2007). Pediatrician perspectives on children’s access to mental health services: consequences and potential solutions. Adm Policy Ment Health Ment Health Serv Res.

[20] Cole A, Kim H, Lotz K, Munson MR (2015). Exploring the perceptions of workers on young adult mental health service (dis)engagement. Soc Work Ment Health.

[21] Riege A, Lindsay N (2006). Knowledge management in the public sector: stakeholder partnerships in the public policy development. J Knowl Manag.

[22] Monterrosa EC, Campirano F, Tolentino Mayo L, Frongillo EA, Hernández Cordero S, Kaufer-Horwitz M (2013). Stakeholder perspectives on national policy for regulating the school food environment in Mexico. Health Policy Plan.

[23] Cargo M, Mercer SL (2008). The value and challenges of participatory research: strengthening its practice. Annu Rev Public Health.

[24] Mathison S (1988). Why triangulate?. Educ Res.

[25] Kane M, Trochim W (2007). Concept mapping for planning and evaluation.

[26] Southern D, Batterham R, Appleby N, Young D, Dunt D, Guibert R (1999). The concept mapping method: an alternative to focus group inquiry in general practice. Aust Fam Phys.

[27] Kane M, Rosas S (2018). Conversations about group concept mapping: applications, examples, and enhancements.

[28] Firth CL, Stephens ZP, Cantinotti M, Fuller D, Kestens Y, Winters M (2021). Successes and failures of built environment interventions: using concept mapping to assess stakeholder perspectives in four Canadian cities. Soc Sci Med.

[29] Jackson KM, Trochim WMK (2002). Concept mapping as an alternative approach for the analysis of open-ended survey responses. Organ Res Methods.

[30] Vives-Cases C, Goicolea I, Hernández A, Sanz-Barbero B, Davó-Blanes M, La Parra-Casado D (2017). Priorities and strategies for improving Roma women’s access to primary health care services in cases on intimate partner violence: a concept mapping study. Int J Equity Health.

[31] Bronfenbrenner U (1979). The ecology of human development experiments by nature and design.

[32] Andersen R (1968). A behavioral model of families’ use of health services.

[33] Durbin J, Goering P, Streiner DL, Pink G (2006). Does systems integration affect continuity of mental health care?. Adm Policy Ment Health Ment Health Serv Res.

[34] Kringos DS, Boerma WG, Bourgueil Y, Cartier T, Hasvold T, Hutchinson A (2010). The european primary care monitor: structure, process and outcome indicators. BMC Fam Pract.

[35] Levesque JF, Harris MF, Russell G (2013). Patient-centred access to health care: conceptualising access at the interface of health systems and populations. Int J Equity Health.

[36] Kidia K, Machando D, Mangezi W, Hendler R, Crooks M, Abas M (2017). Mental health in Zimbabwe: a health systems analysis. Lancet Psychiatry.

[37] Thomée S, Malm D, Christianson M, Hurtig AK, Wiklund M, Waenerlund AK (2016). Challenges and strategies for sustaining youth-friendly health services—a qualitative study from the perspective of professionals at youth clinics in northern Sweden. Reprod Health.

[38] Gulliver A, Griffiths KM, Christensen H (2010). Perceived barriers and facilitators to mental health help-seeking in young people: a systematic review. BMC Psychiatry.

[39] Radovic A, Reynolds K, McCauley HL, Sucato GS, Stein BD, Miller E (2015). Parents’ role in adolescent depression care: primary care provider perspectives. J Pediatr.

[40] Boege I, Herrmann J, Wolff JK, Hoffmann U, Koelch M, Kurepkat M (2018). CCSchool: a multicentre, prospective study on improving continuum of care in children and adolescents with mental health problems associated with school problems in Germany. BMC Health Serv Res.

[41] Macková J (2022). Families at risk and the role of the care system.

[42] Paclikova K, Dankulincova Veselska Z, Filakovska Bobakova D, Palfiova M, Madarasova GA (2018). What role do family composition and functioning play in emotional and behavioural problems among adolescent boys and girls?. Int J Public Health.

